# Cardioprotective Effect of Licochalcone D against Myocardial Ischemia/Reperfusion Injury in Langendorff-Perfused Rat Hearts

**DOI:** 10.1371/journal.pone.0128375

**Published:** 2015-06-09

**Authors:** Xuan Yuan, Hai-tao Niu, Peng-long Wang, Jie Lu, Hong Zhao, Shi-han Liu, Qiu-sheng Zheng, Chang-gui Li

**Affiliations:** 1 The Affiliated Hospital of Qingdao University, Qingdao, 266003, Shandong, China; 2 Binzhou Medical College, Yantai, 264000, Shandong, China; 3 Key Laboratory of Xinjiang Endemic Phytomedicine Resources, Ministry of Education, School of Pharmacy, Shihezi University, Shihezi, 832002, Xinjiang, China; Thomas Jefferson University, UNITED STATES

## Abstract

Flavonoids are important components of ‘functional foods’, with beneficial effects on cardiovascular function. The present study was designed to investigate whether licochalcone D (LD) could be a cardioprotective agent in ischemia/reperfusion (I/R) injury and to shed light on its possible mechanism. Compared with the I/R group, LD treatment enhanced myocardial function (increased LVDP, d*p*/d*t*_max_, d*p*/d*t*_min_, HR and CR) and suppressed cardiac injury (decreased LDH, CK and myocardial infarct size). Moreover, LD treatment reversed the I/R-induced cleavage of caspase-3 and PARP, resulting in a significant decrease in proinflammatory factors and an increase in antioxidant capacity in I/R myocardial tissue. The mechanisms underlying the antiapoptosis, antiinflammation and antioxidant effects were related to the activation of the AKT pathway and to the blockage of the NF-κB/p65 and p38 MAPK pathways in the I/R-injured heart. Additionally, LD treatment markedly activated endothelial nitric oxide synthase (eNOS) and reduced nitric oxide (NO) production. The findings indicated that LD had real cardioprotective potential and provided support for the use of LD in myocardial I/R injury.

## Introduction

Cardiovascular diseases are the main causes of human disability and mortality worldwide [1]. Coronary artery ischemia/reperfusion (I/R) injury is known to occur during the restoration of coronary blood flow after a period of myocardial ischemia and triggers myocardial cell injury and necrosis [2]. I/R injury is an intricate process that implicates many mechanisms. In addition, apoptosis, inflammation and oxidative damage play important roles in I/R injury progression [3–5]. Additionally, antiapoptosis, antiinflammation, and antioxidation have been reported to protect the heart from I/R injury, offering additional evidence that apoptosis, inflammation and oxidative injury are involved in I/R injury [6, 7]. Hence, the modulation of apoptosis, inflammation and oxidative damage and of related cascade responses is considered a crucial therapeutic strategy for treating cardiovascular disease.

**Fig 1:**
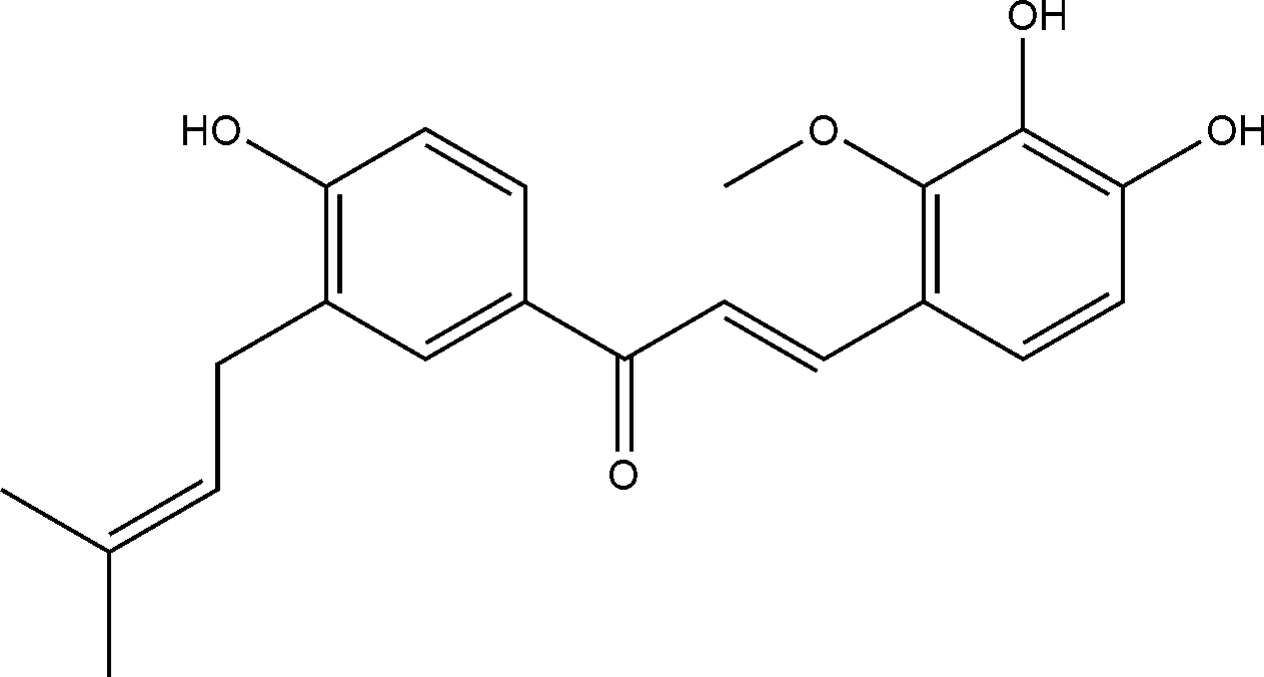
Chemical structure of licochalcone D.

Flavonoids are the most potent and versatile biologically active compounds in edible plants and have beneficial influences on the cardiovascular system, including anti-hypertensive [8–11], anti-atherosclerotic [12–14], and anti-platelet effects [15]. Indeed, experimental and epidemiological studies have suggested that this phytochemical class affects circulatory functions [16, 17]. Additionally, the antioxidant property of flavonoids is responsible for preventing some cardiovascular diseases [18–20]. More importantly, flavonoids produced cardioprotective effects in experimental models of myocardial ischemia/reperfusion (I/R) injury [21–27]. Licochalcone D (LD, Fig 1), which is flavonoid compound primarily existing in the root of *Glycyrrhiza inflata*, possesses antioxidant and antiinflammatory properties [28–30]. Furthermore, LD exerts its antiinflammatory effect by suppressing mast cell degranulation and LPS-induced phosphorylation of NF-κB [31, 32]. However, the mechanism by which this component exerts effects on I/R injury remains unclear. Therefore, the purpose of the present study was to investigate whether LD might prevent myocardial I/R injury and to determine the potential mechanisms involved.

## Materials and Methods

Male Sprague-Dawley (SD) rats (250–280 g) were obtained from Xinjiang Medicine University Medical Laboratory Animal Center (SDXK (Xin) 2011–004), Xinjiang Medical University, Xinjiang, China. The rats were maintained under standard animal care conditions (22 ± 3°C and 60% humidity), with a commercial standard mouse diet (Shihezi University Laboratory Animal Center, Xinjiang, China) and water ad *libitum*. And all experimental procedures were approved by the Ethics Committee of Shihezi University.

### Isolated rat heart preparation

The male SD rats (250–280 g) were anesthetized by an intraperitoneal injection of 60 mmol/L chloral hydrate (0.35 g/kg). To anticoagulate, 250 U/kg of heparin were administered intraperitoneally to the rats. Thoracic surgery was performed to remove the heart. Hearts were excised quickly and immersed in ice-cold Krebs-Henseleit buffer [33] (118 mM NaCl, 1.2 mM KH_2_PO_4_, 4.7 mM KCl, 1.2 mM CaCl_2_, 1.2 mM MgSO_4_, 24.9 mM NaHCO_3_ and 11.1 mM glucose, pH 7.4) comprised of 95% O_2_ 5% CO2 to suppress contraction and reduce oxygen consumption. Immediately, the heart was mounted on Langendorff's apparatus and equilibrated with a gas mixture bubbled with 95% O_2_, 5% CO_2_ at 37°C. The above course was finished within 2 min. A water-filled latex balloon, coupled to a pressure transducer (Statham), was inserted into the left ventricular cavity through the left auricle, and the volume of the balloon was adjusted to maintain a stable left ventricular end-diastolic pressure of 5–12 mmHg during initial equilibration [16].

### Measurement of heart hemodynamic parameters

The following functional parameters were continuously monitored with a computer-based data acquisition system (PowerLab/4S with Chart 5 software, AD Instruments, Gladstone, Australia): left ventricular systolic pressure (LVSP), left ventricular end-diastolic pressure (LVEDP), left ventricular developed pressure (LVDP, LVDP = LVSP-LVEDP), maximum rise/down velocity of left intraventricular pressure (d*p*/d*t*_*max*_ and d*p*/d*t*_*min*_), coronary flow (CF) and heart rate (HR). The heart effluents were collected at 1 min intervals to determine the coronary flow (CF). The recovery of LVDP, d*p*/d*t*_*max*_, d*p*/d*t*_*min*_, CF and HR were expressed as the percent of 1 minute before ischemia.

### Experimental groups

**Fig 2:**
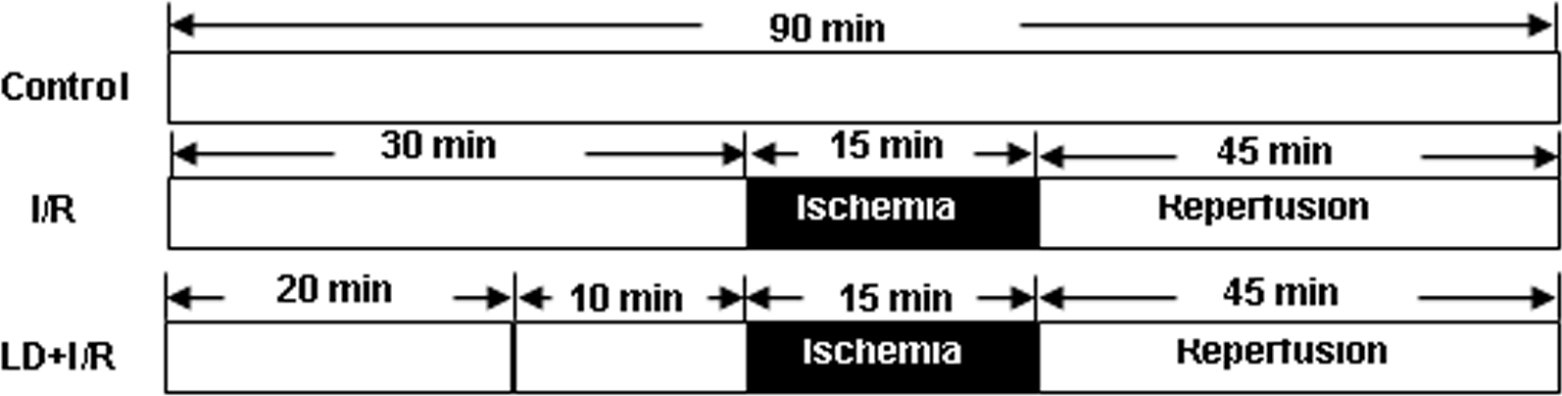
Experimental protocol

The isolated rat hearts were randomly divided into three groups (n = 8): control group (Sham), I/R group and I/R with LD. Control group hearts were perfused for the 90 min stabilization period. I/R group hearts were stabilized for 30 min, and then global ischemia (no flow) and reperfusion were subjected to 15 min and 45 min, respectively. Hearts in LD group hearts were stabilized for 20 min, instead K-H buffer with indicated concentration of LD, global ischemia and reperfusion were established for 10 min, 15 min and 45 min, respectively (Fig 2). Left ventricular (LV) function was measured at baseline, just prior to ischemia and 45 min after reperfusion, because the recovery of LV function after reperfusion reached a maximum within 45 min.

### Enzyme activity assays

Lactate dehydrogenase (LDH) and creatine kinase (CK) activity was measured to assess the extent of myocardial cell injury [34]. Briefly, samples were collected from the coronary effluent at the end of the experiment, and the activities of LDH and CK were assayed using LDH and CK kits (Nanjing Jiancheng Bioengineering Institute, Nanjing, China).

### Determination of myocardial infarct size

The myocardial infarct size (INF) was determined by means of a double-staining technique and was evaluated by a digital imaging system described previously [35]. At the end of the reperfusion, the heart was removed, washed in phosphate buffered saline, frozen (−20°C, 30min), and then sliced into 1mm sections perpendicularly along the long axis from apex to base. The slices were incubated in 1% triphenyltetrazolium chloride (TTC, Biodee, Beijing, China) in pH 7.4 buffer at 37°C for 10–15 min, fixed in 10% formaldehyde solution, and imaged using a digital camera. Then the infarct (white), non-infarct (red) areas were measured an Image-Pro Plus 7.0 (Media Cybernetics, Wyoming, USA). The myocardial infarct size was represented as a percentage of infarct area (INF) over total area of sections (LV, left ventricle area) × 100%.

### Nitrate plus nitrite (NOx) measurement

NOx, the stable end product of NO, has been tested as an index of bioavailability of NO. The level of myocardial NOx was assessed using a commercial kit (Nanjing Jiancheng Bioengineering Institute, Nanjing, China). NOx was designated as μmol/g protein. Its concentration was determined at an optical density of 550 nm in a spectrophotometric method. Protein determination was carried out according to the Bradford method [36].

### Oxidative stress assay

After 45 min of the perfusion, hearts were harvested and frozened at -70°C for following experiments. The hearts were grinded by a liquid nitrogen-chilled tissue pulverizer, weighed the cardiac tissues, and then homogenized in bufer and centrifuged using homogenizer. The superoxide dismutase (SOD), malondialdehyde (MDA) and glutathione/glutathione disulide (GSG/GSSH) concentrations were measured with or without the inhibitors (MK0026, PDTC, or SB203580) according to the instruction of the kits (Nanjing Jiancheng Bioengineering Institute, Nanjing, China). The levels of MDA were expressed as nanomole MDA per milligram protein.

### Inflammatory cytokines measurement

To investigate the LD’s role in I/R-induced inflammation response, the levels of proinflammatory cytokines (IL-6, TNF-α and CRP) were detected with or without the inhibitors (MK0026, PDTC, or SB203580) according to the instruction of the Rat Interleukin 6 ELISA Kit, Rat Tumor Necrosis Factor α ELISA Kit, and Rat CRP/C-Reactive Protein ELISA Kit (Sigma-Aldrich, St. Louis, MO, USA)

### Determination of caspase-3, PARP, eNOS, iNOS expression, iNOS, total or phosphorylaed AKT, NF-κB/p65 and p38 MAPK by Western blot assay

Frozen myocardial tissue samples were ground with a mortar and pestle, and subsequently placed into a tissue grinder with lysis buffer. The protein concentration was determined with a Bio-Rad protein assay (Bio-Rad Laboratories, Hercules, CA, USA). The lysate samples were separated by electrophoresis on sodium dodecyl sulfate-polyacrylamide gel electrophoresis (SDS-PAGE), nitrocellulose membranes (Amersham Biosciences, New Jersey, USA), and blocked with 5% nonfat milk in Tris-buffered saline with Tween (TBST) for 2 h at room temperature. Membranes were incubated with an antibody against cleaved caspase-3, cleaved-PARP, and phosphorylated eNOS (Cell Signaling Technology, MA, USA), or iNOS (BD Bio-science Laboratories, CA, USA), total or phosphorylaed AKT, NF-κB/p65 and p38 MAPK (Sigma-Aldrich, MO, USA) in 5% milk/TBST at 4°C overnight. The membrane was then washed with TBST and incubated with horseradish peroxidase-conjugated antibody (Cell Signaling Technology) for 1 h at room temperature. The blots were developed with an enhanced chemiluminescence detection kit (Thermo, NY, USA) and were exposed on Kodak radiographic film. The immunoblotting was visualized with EC3 Imaging System, and analyzed with Vision Works LS software (UVP, CA, USA).

### Terminal DNA breakpoints in situ 3-hydroxy end labeling (TUNEL) assay

The TUNEL staining kit was purchased from Roche Group (Basel, Switzerland). According to the manufacturer’s instruction, the tissue sections were deparaffinized by immersing slides in xylene, and rehydrated by sequentially immersing the slides through graded ethanol washes. The sections were treated with protease K (20 ug/mL) for 15 min, then were immersed in TUNEL reaction mixture for 60 min at 37°C in a humid chamber, and incubated with converter-POD was used to incubate the slides for 30 min at 37°C. The slides were observed by optical microscopy.

### Statistical analysis

Data were presented as mean±SD from at least six independent experiments and statistical comparisons were evaluated with One-way ANOVA and followed by Student's *t*-test. Differences were considered significant at *p* < 0.05. Analyses were performed using the SPSS 17 software (SPSS Inc., Chicago, IL, USA, 2008).

## Results

### LD improved the recovery of I/R-altered cardiac function

**Fig 3:**
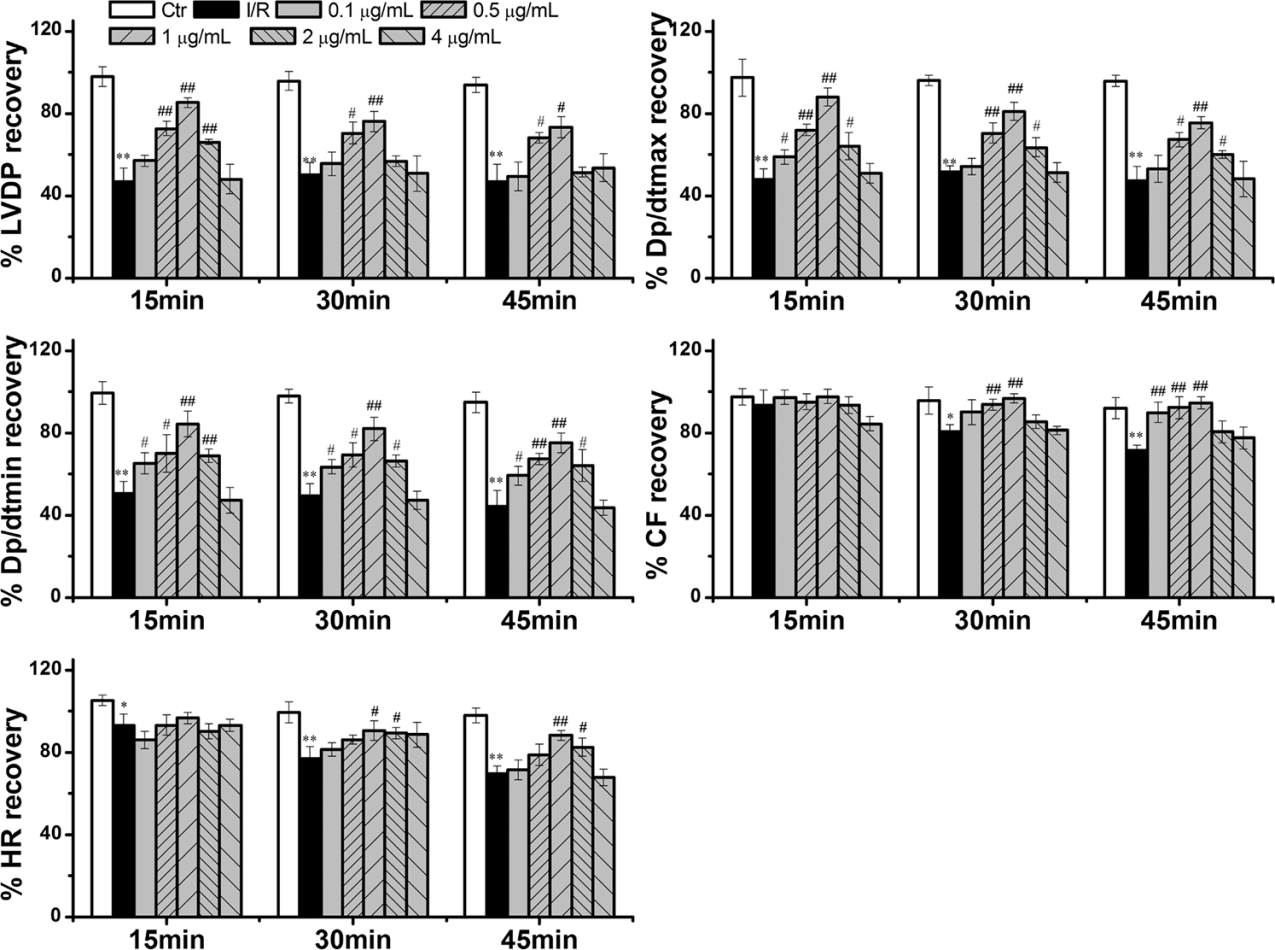
Effect of LD on cardiac function in rats subjected to I/R (n = 8). The three time points (15 min, 30 min and 45 min) represente the the time points after the start of reperfusion. ^*^ *P*<0.05, ^**^ *P*<0.01 compared with control group; ^*#*^ *P*<0.05, ^*##*^ *P*<0.01 compared with I/R group.

The effects of LD (0.1, 0.5, 1, 2 and 4 μg/mL) on hemodynamic parameters (LVDP, ±d*p*/d*t*_max_, CF and HR) in the control group, I/R group and LD-treated group hearts were shown in Fig 3. The hearts subjected to 15 min of ischemia, followed by 45 min of reperfusion, showed a significant decrease in the values of LVDP (49.38%), d*p*/d*t*_max_ (47.30%) d*p*/d*t*_min_ (44.46%), CF (71.40%) and HR (69.47%) after 45 min of reperfusion. Compared with unprotected I/R hearts, LD pre-treatment increased the cardiac functional index, and 1 μg/mL of LD significantly improved the values of LVDP, d*p*/d*t*_max_, d*p*/d*t*_min_, CF and HR but had little effect on cardiac function (S1 Table).

### LD decreased myocardial injury in I/R rats

**Fig 4:**
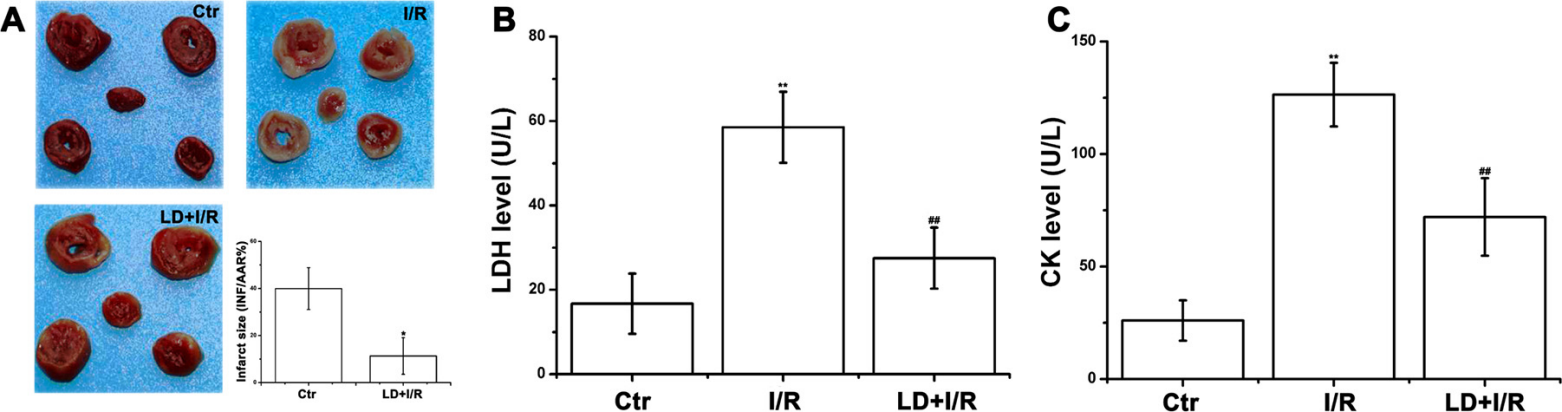
LD reduced I/R-induced myocardial injury. (A) The myocardial infarct size. (B) The level of LDH. (C) The level of CK. ^**^ *P*<0.01 compared with control group; ^*##*^ *P*<0.01 compared with I/R group.

Considering the significant cardioprotective effects of LD at 1 μg/mL, this concentration was chosen for the subsequent assays. Myocardial infarct size (INF), lactate dehydrogenase (LDH) and creatine kinase (CK) levels were measured to examine whether LD might reduce myocardial injury. As shown in Fig 4, the myocardial INF (39.94±8.89%), LDH leakage (58.5±8.43) and CK (126.36±14.13) markedly increased in the hearts of rats in the I/R group after 15 min of ischemia, followed by 45 min of reperfusion, compared to controls. In contrast, LD pre-treatment significantly reduced the I/R-induced increase in myocardial INF (11.30±7.78%), LDH (27.5±7.26) and CK (72.00±17.24) release in rat heart.

### LD suppressed myocardial apoptosis

**Fig 5:**
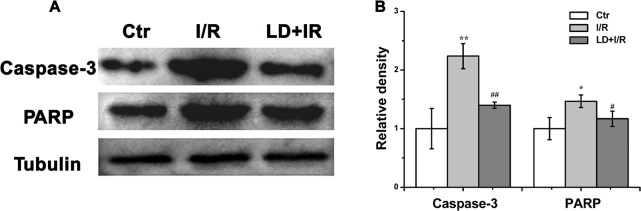
LD suppressed myocardial apoptosis. (A) Cleaved caspase-3 and PARP expressions in cardiac tissue were analyzed via Western blot. (B) Quantitative analysis of cleaved caspase-3 and PARP protein levels. ^*^ *P*<0.05, ^**^ *P*<0.01 compared with control group; ^*#*^ *P*<0.05, ^*##*^ *P*<0.01 compared with I/R group.

Apoptosis is the major form of cell death after a short period of ischemia that is followed by reperfusion; therefore, we investigated the effects of LD treatment on myocardial apoptosis-related proteins (caspase-3 and PARP) in I/R cardiac tissue. As shown in Fig 5, I/R induced increased activation of apoptosis, as illustrated by the cleaved caspase-3 protein expression, which is associated with a 1.47-fold increase in cleaved PARP, compared with the control group. LD treatment substantially reduced this I/R-induced cleavage of both caspase-3 and PARP.

### LD treatment alleviated inflammation in I/R cardiac tissue

**Fig 6:**
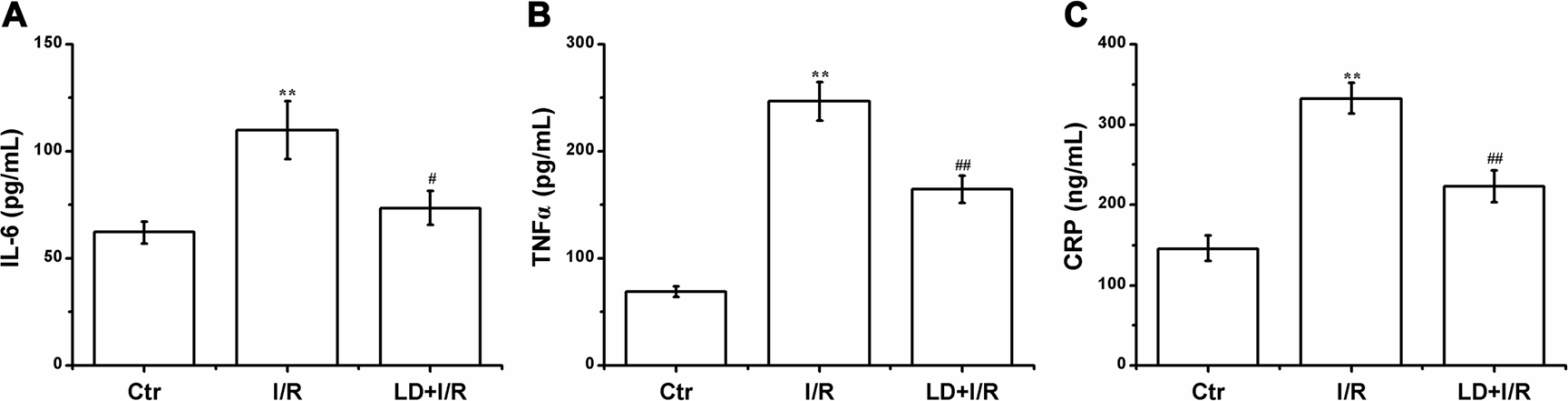
Effect of LD on inflammatory cytokines in rats subjected to I/R. (A) The level of IL-6. (B) The expression of TNF α. (C) The expression of CRP. ^**^ *P*<0.01 compared with control group; ^*#*^ *P*<0.05, ^*##*^ *P*<0.01 compared with I/R group.

Previous studies have indicated that LD has antiinflammation effect and that the inflammatory response participates in I/R-induced cardiac injury; thus the effects of LD on inflammatory cytokines (IL-6, TNF-α and CRP) were also detected in the ischemic heart. I/R injury significantly increased the IL-6 expression level and activated TNF-α and CRP expression. The IL-6 expression level in the LD treatment group (62.12±5.05 pg/mL) was markedly lower than that in the I/R group (109.83±13.62 pg/mL). Compared with the I/R group, the activities of TNF-α and CRP significantly decreased (247.11±18.05 pg/mL *vs*. 164.39±12.44 pg/mL) and (332.66±19.40 pg/mL *vs*. 223.05±19.39 pg/mL), respectively (Fig 6).

### LD treatment attenuated oxidative stress in I/R cardiac tissue

**Fig 7:**
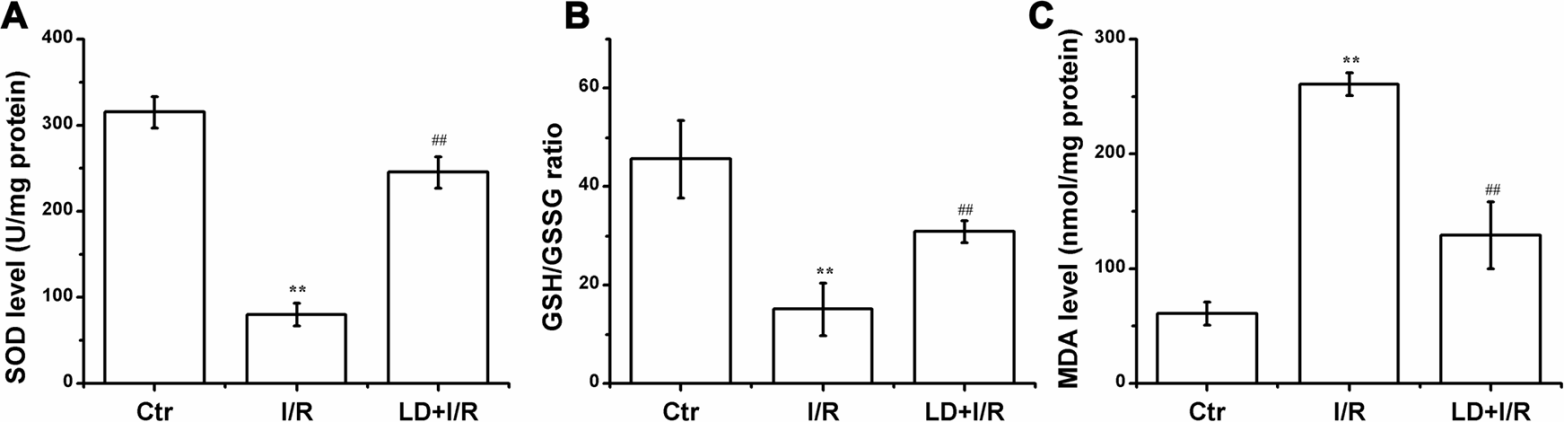
Effect of LD on the activity of SOD, the ratio of GSH/GSSG, and the content of MDA. (A) The activity of SOD. (B) The ratio of GSH/GSSG. (C) The content of MDA. ^**^ *P*<0.01 compared with control group; ^*##*^ *P*<0.01 compared with I/R group.

Considerable evidence demonstrated that overproduction of ROS and resultant oxidative stress play a causative role in I/R-induced cardiac injury and apoptosis. Therefore, we examined SOD activity, MDA contents, and the GSH/GSSG ratio. Decreases in SOD activity and in the GSH/GSSG ratio and an increase in MDA content were noted following I/R, and these changes were significantly inhibited by treatment with 1 μg/mL of LD (Fig 7).

### Effect of LD treatment on apoptosis-, inflammation- and oxidative stress-related proteins in I/R cardiac tissue

**Fig 8:**
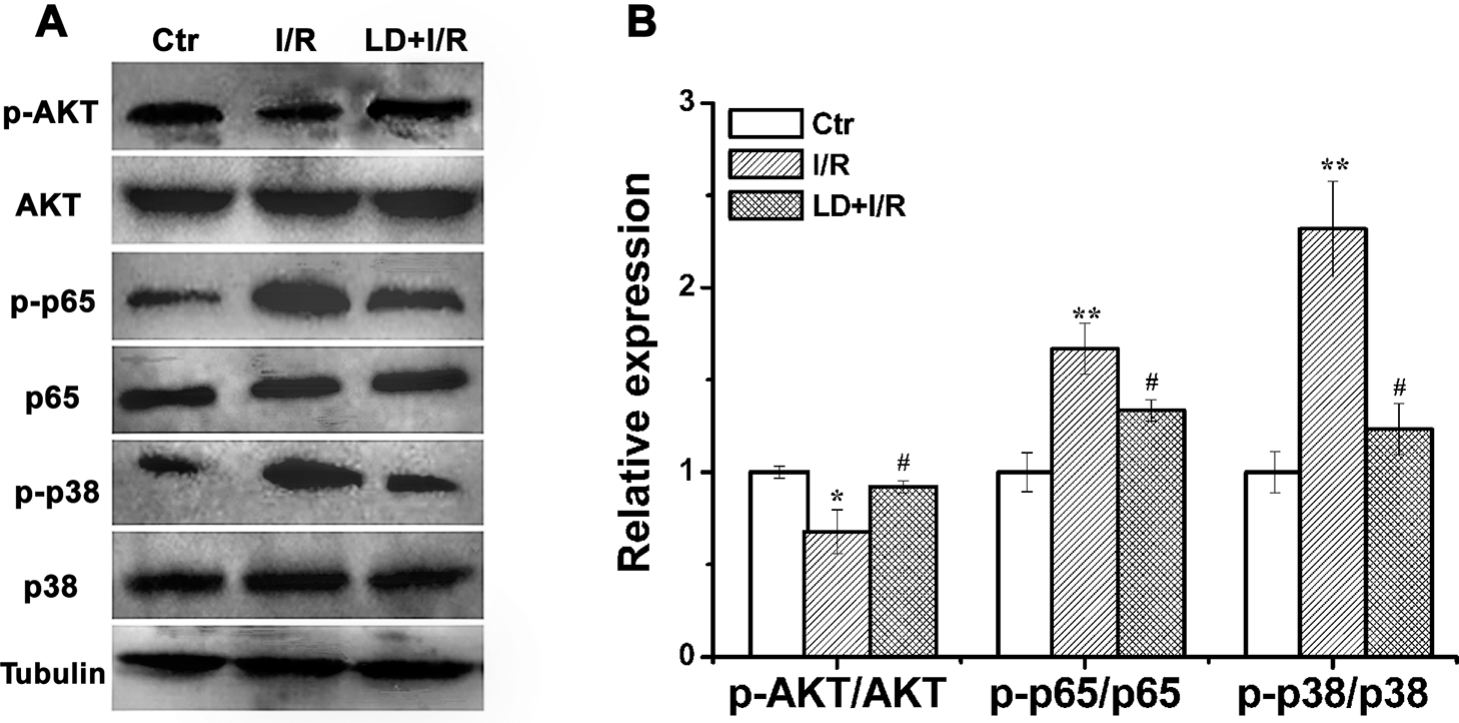
Effect of LD on the apoptosis, inflammation and oxidative stress related proteins expressions were measured. (A) The total and phosphorylaed AKT, NF-κB/p65 and p38 MAPK expressions in cardiac tissue were analyzed *via* Western blot. (B) Quantitative analysis of these proteins levels. ^*^ *P*<0.05,^**^ *P*<0.01 compared with control group; ^*#*^ *P*<0.05 compared with I/R group.

**Fig 9:**
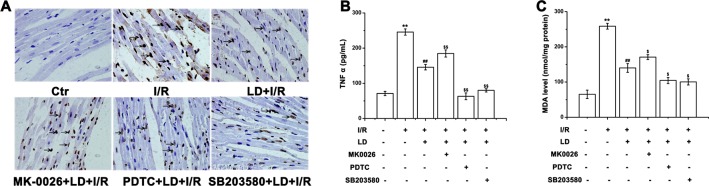
LD inhibited the apoptosis, inflammation and oxidative stress *via* activation of AKT and inactivation of NF-kB/p65 and p38 MAPK. The hearts were pretreated with inhibitors AKT inhibitor (MK-2206, 10 μM), NF-κB/p65 inhibitor (PDTC, 20 μM) or p38 MAPK inhibitor (SB203580, 10 μM) plus LD (1 μg/mL) for 10 min before exposure to ischemia for 15 min, and then reperfusion for 45 min. (A) Apoptotic cells were detected by TUNEL assays. (B) The expression of TNF α was analyzed. (C) The content of MDA was measured. ^**^ *P*<0.01 compared with control group; ^*##*^ *P*<0.01 compared with I/R group; ^*$*^ *P*<0.05, ^*$$*^ *P*<0.01 compared with LD+I/R.

To illuminate the mechanism of the cardioprotective effects of LD, the expression of apoptosis-, inflammation- and oxidative stress-related proteins was measured *via* Western blot analysis. Compared with the I/R group, LD treatment significantly increased the relative levels of phosphorylated AKT but decreased the relative levels of NF-κB/p65 and the phosphorylation of p38 MAPK (Fig 8). Experiments were performed using a series of inhibitors, namely, AKT inhibitor (MK-2206, 10 μM), NF-κB/p65 inhibitor (PDTC, 20 μM) and p38 MAPK inhibitor (SB203580, 10 μM), to obtain additional evidence to support this conclusion. As summarized in Fig 9, LD treatment markedly improved I/R-induced myocardial damage, as evidenced by reductions in TUNEL-positive staining (a marker of apoptosis); the TNF-α level (a marker of inflammation) and MDA content (a marker for oxidative injury). Moreover, compared with the LD pre-treatment group, combined LD treatment with MK-2206 (AKT inhibitor) aggravated I/R-induced apoptosis, the TNF-α level and MDA content. In contrast, combined LD treatment with PDTC (NF-κB/p65 inhibitor) or SB203580 (p38 MAPK inhibitor) eased these effects. Taken together, these results suggest that LD may protect against myocardial I/R injury *via* the modulation of AKT, NF-κB/p65 and p38 MAPK-mediated signaling pathways.

### LD treatment decreased NOx content and inhibited eNOS phosphorylation in I/R cardiac tissue

**Fig 10:**
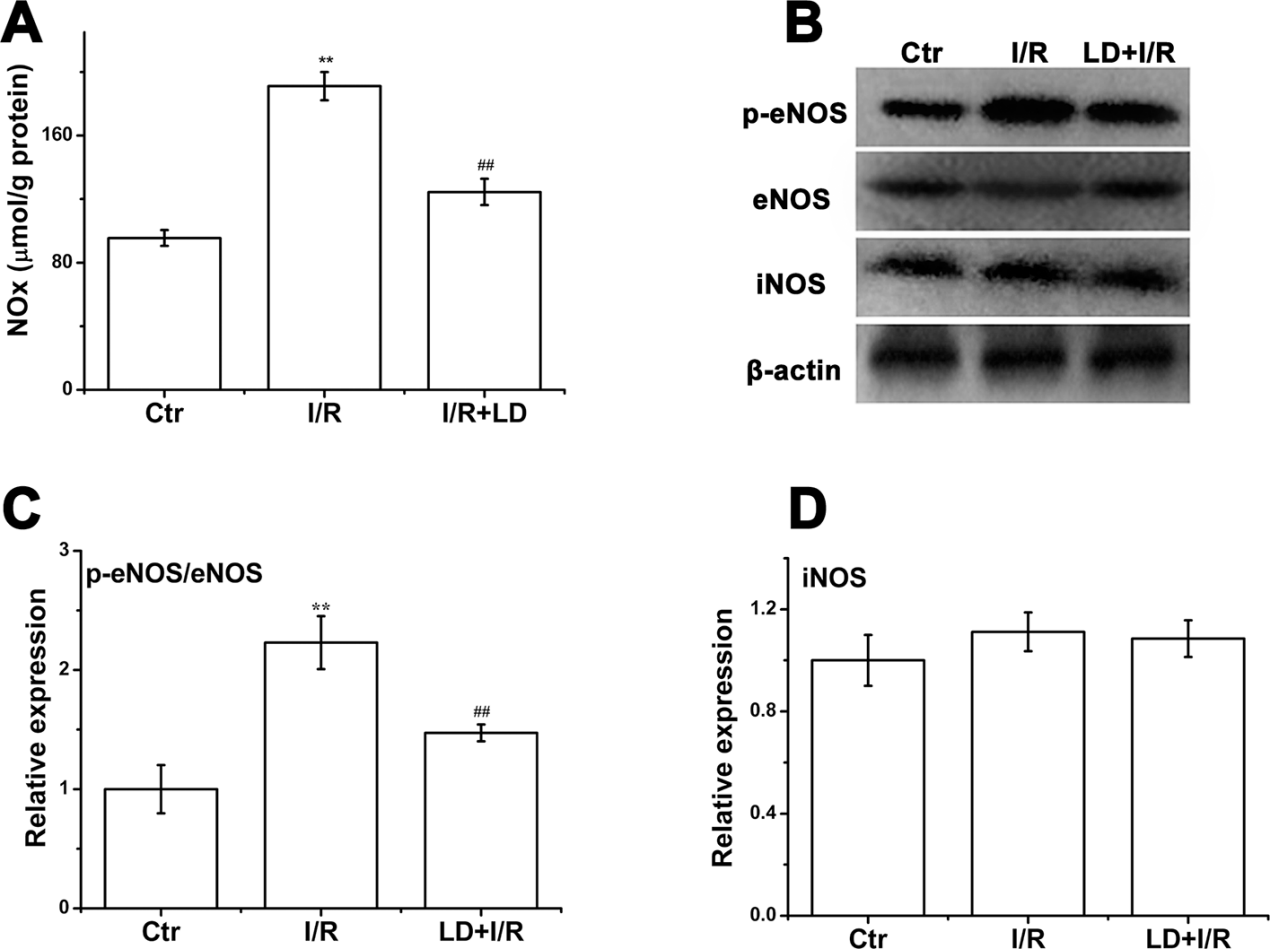
Effect of LD on NO production, iNOS protein expression, and eNOS phosphorylation. (A) The level of NO production. (B) The protein expression of total eNOS, phosphorylaed eNOS, and iNOS and were analyzed *via* Western blot. (C) Quantitative analysis of phosphorylaed eNOS/ total eNOS protein level. (D) Quantitative analysis of iNOS protein level. ^**^ *P*<0.01 compared with control group; ^*##*^ *P*<0.01 compared with I/R group.

The previous experiment indicated that LD inhibited the LPS-induced nitric oxide (NO) production, and the vital function of NO in cardiovascular disease has been demonstrated previously; thus, we explored the role of NO production in LD-mediated cardiac protection. As shown in Fig 10A, LD treatment resulted in a decrease in NOx content. The eNOS and iNOS levels were also measured due to their importance in NO production in the heart. The results indicated that LD markedly decreased eNOS phosphorylation, whereas no significant difference in iNOS expression was observed among the different groups (Fig 10B, 10C and 10D).

## Discussion

Several important observations were made in our present experiments. Firstly, we observed for the first time that LD treatment markedly attenuated the I/R-induced cardiomyocyte apoptosis, inflammatory response and oxidative damage. Additionally, cardiac functional recovery of LD declined 2 μg/mL of LD may be relevant to the low of antioxidant and antiinflammatory capacities (S1 Fig). Secondly, these effects were related to the activation of the AKT pathway and to the inactivation of p38 MAPK, JNK1/2 and NF-κB/p65 pathways. Most importantly, LD treatment inhibited I/R-mediated NO overproduction and eNOS activity.

Myocardial ischemia/reperfusion (I/R) injury leads to heart dysfunction and to cardiomyocyte apoptosis [5, 37]. Several studies have clarified the deleterious effects of I/R injury. We also observed myocardial dysfunction and apoptosis in the present study, as evidenced by the changes in cardiac functions, myocardial injury (myocardial INF, CK and LDH levels), and induced apoptosis (activated the caspase-3 and PARP) after myocardial I/R injury. The improved hemodynamic parameters (Fig 3), decreased INF, CK and LDH levels (Fig 4), and inhibited cardiomyocyte apoptosis (Fig 5 and S1 Fig) convincingly favored the cardioprotective effects of LD on the heart after I/R injury.

Inflammation is involved in I/R-induced cardiac injury. Inflammatory cell infiltration is the early step of cardiac I/R injury, and the activation of NF-κB/p65 is a major pathway that modulates cardiac inflammation and injury [38, 39]. LD significantly inhibited mast cell degranulation in a previous study, suggesting that LD has a potential inhibitory effect on inflammation [31]. Moreover, experimental evidence suggested that the antiinflammatory effect of LD was associated with the inhibition of the NF-κB signaling pathway [32]. However, the effect of LD on myocardial I/R-induced inflammation is unidentified. The results of the present study showed that LD treatment markedly inhibited the I/R-induced activities of cytokines and proinflammatory factors, including IL-6, TNF-α, CRP and NF-κB/p-p65 (Figs 6 and 9 and S1 Fig). Based on these observations, it suggested that LD is a promising natural product for administration of I/R-induced cardiac inflammatory response.

Oxidative stress is thought to be the crucial event in I/R injury [3, 4]. Consequently, inhibiting oxidative stress is considered a viable approach for treating I/R-induced cardiac injury [40, 41]. LD, possesses antioxidant properties, could scavenge superoxide anion and DPPH radicals, protecting biological systems against oxidative damage produced by Fe(III)-ADP/NADH [28]. In the present study, we demonstrated that LD treatment significantly reduced the MDA (a critical component of NADPH oxidase) content in I/R rats and improved the I/R-induced reduction of total antioxidant capacity (SOD activity) and the GSH/GSSG ratio (Fig 7 and S1 Fig). Thus, these findings suggested that LD exhibits a cardioprotective role by regulating oxidative stress.

Nitric oxide (NO) itself is nontoxic and does not result in significant tissue injury [42]. It is essential for normal heart function *via* its vasodilator, antiplatelet, and antineutrophil actions. however, the role of NO in myocardial injury and dysfunction remains under dispute. Recent study has demonstated that the NO level of heart tissues is within a low range at baseline and enhances during ischemia [43]. Although a slight increase in NO content may be cardioprotective, a large increase seems to be detrimental because of the ONOO^-^ formation [44, 45]. Thus, it could be hypothesized that reducing NO production and subsequently decreasing the levels of nitro-oxidative stress could protect the heart from I/R injury. In our present study, LD stimulated decreased eNOS phosphorylation and NO production after 45 of reperfusion; however, no obvious change was observed in iNOS expression after LD treatment (Fig 10). These data suggested that LD might inhibit eNOS activity and NO production, thus exerting cardioprotection. However, the lack of a significant difference in iNOS expression after LD treatment might due to the limitation of reperfusion because the evidence indicated that iNOS expression was detectable at 3 h of reperfusion but not at 1 h of reperfusion [46, 47].

Several signaling pathways are related to cardioprotective effects, including AKT, p38 MAPK, and NF-κB [5, 48, 49]. AKT is a key factor that strengthens cell survival by inhibiting caspase-activated apoptosis [5, 50]. JNK (c-Jun N-terminal kinase) and p38 MAPK, which are members of the mitogen-activated protein kinase (MAPK) family, play important roles in response to I/R injury and are involved in cardiomyocyte apoptosis, the inflammatory response and oxidative injury [51–53]. For example, AKT activation is essential for cardiomyocyte survival, whereas p38 MAPK suppression ameliorates cardiac injury [54]. In this study, the results showed that LD treatment led to the activation of AKT and to the inhibition of p38 MAPK and NF-κB/p65 signaling pathways after I/R injury (Fig 8). Moreover, treatment with specific inhibitors further demonstrated that these signaling pathways participate in the LD-mediated cardioprotective effect (Fig 9). Thus, LD protects against I/R-induced myocardial injury possibly through modulating multiple signaling pathways.

## Conclusions

In conclusion, the results from our experiments suggest that LD treatment may decrease cardiac infarct size and inhibit apoptosis, inflammation and oxidative stress. Additionally, these effects might be associated with the activation of the AKT pathway and with the inactivation of the p38 MAPK and NF-κB/p65 pathways in the I/R-injured heart; however, this study still has some limitations. (1) Our data demonstrate that LD treatment suppresses eNOS phosphorylation and NO production; however, determining the effect of LD treatment on iNOS expression requires further investigation. (2) The present study demonstrated that LD inhibits oxidative stress, which is required for recovery from cardiac I/R injury. However, whether LD specifically targets the pathological source of ROS (NADPH oxidases, mitochondria and others) to exert its antioxidant effect remains unknown. (3) Determining the pathway of LD against I/R-injured heart and further clarifying the potential relationship of the pathways.

## Supporting Information

S1 Fig Effect of LD (2 μg/mL) on PARP, TNF α and SOD in rats subjected to I/R. (A) Cleaved PARP expression in cardiac tissue was analyzed via Western blot. (B) The expression of TNF α. (C) The activity of SOD. ^**^ *P*<0.01 compared with control group; ^*#*^ *P*<0.05, ^*##*^ *P*<0.01 compared with I/R group.
(TIF)

S1 Table Effect of LD (1 μg/mL) alone on cardiac function in rats subjected to I/R (n = 8). The three time points (15 min, 30 min and 45 min) represente the the time points after the start of reperfusion. *P*>0.05 compared with control group.
(TIF)
