# Supplementary figures and images for: Cardioprotective Effect of Licochalcone D against Myocardial Ischemia/Reperfusion Injury in Langendorff-Perfused Rat Hearts
Source: PLoS One. 2015 Jun 9;10(6):e0128375. doi: 10.1371/journal.pone.0128375 (PMC4461317; doi:10.1371/journal.pone.0128375)

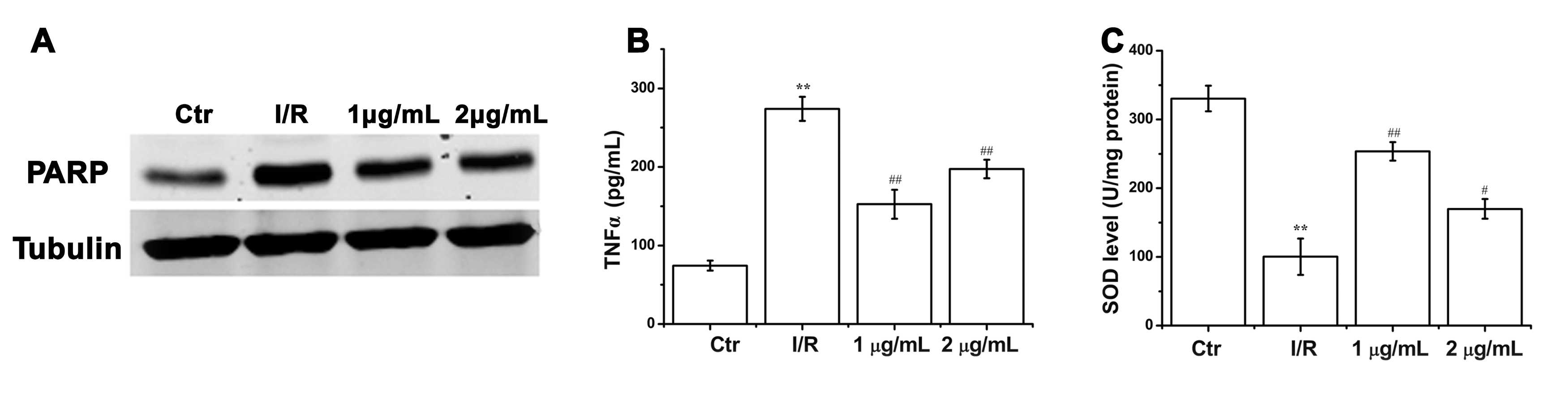

Supplement: S1 Fig — (A) Cleaved PARP expression in cardiac tissue was analyzed via Western blot. (B) The expression of TNF α. (C) The activity of SOD. ** P<0.01 compared with control group; # P<0.05, ## P<0.01 compared with I/R group. (TIF) [file pone.0128375.s001.tif]

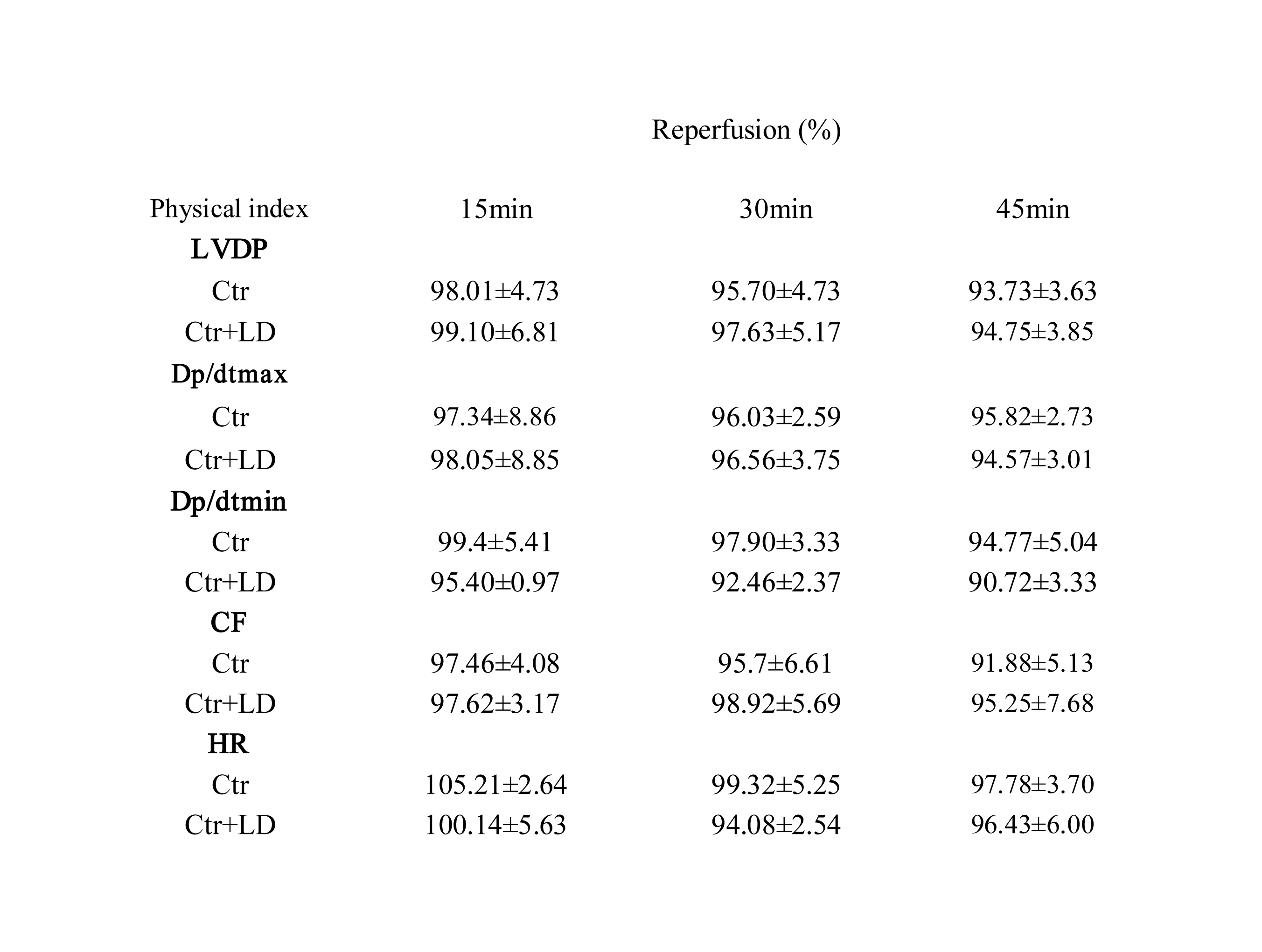

Supplement: S1 Table — The three time points (15 min, 30 min and 45 min) represente the the time points after the start of reperfusion. P>0.05 compared with control group. (TIF) [file pone.0128375.s002.tif]
